# Editorial Department

**Published:** 1884-04

**Authors:** 


### The Bistoury.

ELMIRA, N. Y., APRIL, 1884.

The BISTOURY is published Quarterly, upon the first of April, July, October and January, at Fifty Cents a year in advance.

### Editorial Department.

A DOCTOR FOR MAYOR.—Elmira has just shown its good sense in electing Dr. Henry Flood its mayor. It is a comfort to know that the politicians *can* be, sometimes, defeated, and an intelligent and respectable gentleman occasionally elected to office. Dr. Flood has the necessary vim, courage and executive ability to enforce the laws, and to make us one of the best, if not the very best, mayors the city has ever had.

CONFESSING CRIME—Daily, we read of parties who have committed crimes—as arson, robbery, murder and the like—and who, being hard pressed by the officers of the law, make full confession of their crime. This circumstance has always appeared to me somewhat singular. It would seem that any fellow, scoundrel enough to commit such a crime, would be equally capable of lying about it to the end,—at least one would have more respect for him.

THE POSTAL TELEGRAPH—We see that this company has made a proposition to the United States Senate to transmit messages over its lines at a rate of twenty-five cents for twenty words, and double those rates from the Atlantic to the Pacific. We doubt whether the concern can ever be made popular and to pay at those rates. A telegraph for "postal" purposes should not cost its patrons more than ten cents for a message of twenty words. If this rate cannot be afforded, the American people can get along very well with their present facilities.

CANNED FRUIT—We see there is a bill before the New York legislature seeking to require of manufacturers of canned goods, that they stamp upon every package the date of the canning, and prohibiting the sale of goods over one year old. Should this bill become a law, doubtless the business of canning fruits, etc., would be killed. Would it not be better and more sensible to require of packers a better quality of tin in their cans? It will be noticed that the tin of which these packages are composed is of the thinnest and flimsiest character, the acids from the fruit soon destroying it, permitting the contents of the can to come in contact with the iron, so causing certain salts of iron that have acted in a detrimental manner upon the health of the consumer.

THE SUICIDE MANIA—It seems "to be in the air." One chap who imagines he has, or really has, sufficient cause for terminating his existence, does so in a heroic manner. The circumstance is minutely related in the daily press. A melancholy, diseased, or worried fellow reads it, speculates and comments upon it, until himself is convinced of the feasibility and justness of the undertaking. It is not safe, in these realistic times, to discuss such a matter, particularly by those who have any cause for such an undertaking, because dwelling upon it is apt to finally convince them that suicide is the easiest way out of their trouble. *Some* men are, likely, justifiable in taking their own lives. The majority of those who resort to that method of comfort, are not.

A BAD PRACTICE.—"Those scholars who bring their dinners to school are not allowed to spend the noontime which is left after eating ther lunch, foolishly. They are obliged to spend three-quarters of an hour in study."

So says our daily *Advertiser*. If this is true, if boys and girls are permitted—much less compelled—to study during the noon interval, it is an outrage. Pupils at the academy are taxed to their full physical strength and endurance, during the usually allotted study hours, without permitting them to keep it up when they should be exercising in the open air. Cases can be readily cited of strong, vigorous, young people who have laid the foundation for serious diseases, while pursuing the vigorous course of study and discipline at the academy. If there be truth in the above quoted item, the Board of Education are derelict in their duty if they do not prohibit study during the play hour.

HOME FOR THE AGED.—This worthy society is engineered by a bevy of ladies who have manifested great tact in the management of its affairs. Particularly have they shown eminent ability in conducting entertainments of various kinds, as a means of securing revenue to the exchequer of the Home. Their very first venture in this direction—the Loan Exhibition—in which they realized $2,522.12, was a marvel of success, both as an exhibition and in securing funds. Then, their recent Author's Carnival, showed their skill in conducting theatrical performances, and in training little children in very pleasing and charming acts. The "Mother Hubbard" party, in which the little *wee* people were taught to enact the characters found in Mother Hubbard, and that other wonderful dame, dear in childhood's memory, "Mother Goose," was an example of indomitable energy, perseverance and genius in the training of children, rarely encountered. The three days' entertainment netted the lady managers $1,400. Certainly, no party of men could have done better.

---

HYGIENE IN THE SCHOOLS—And now, our wise legislators have passed a law relating to the public schools of the state of New York, the first two sections of which read as follows:

SECTION 1. Provision shall be made by the proper local school authorities for instructing all pupils in all schools supported by public money, or under state control, in physiology and hygenie, *with special reference to the effects of alcoholic drinks, stimulants and narcotics* upon the human system.

SEC. 2. No certificate shall be granted to any person to teach in the public schools of the State of New York after the first day of January, eighteen hundred and eigthy-five, who has not passed a satisfactory examination in physiology and hygenie, *with special reference to the effects of alcoholic drinks stimulants and narcotics* upon the human system.

Italics ours, calling attention to the absurdity of particularizing the teaching of hygiene with reference to any special subject. Why not instruct the pupils with regard to the consequences of jumping-the-rope three thousand times or so, chewing gum, or the danger that lurks in promiscuous kissing and hugging one's girl too much ?

---

ACCOUNTING FOR SICKNESS—The human species is possessed of numerous peculiar attributes, not the least perplexing of which is, the fondness for determining the origin of disease and volunteering an unsolicited opinion to the sufferer as to the means of cure.

Let an individual be subject of any special ailment, that at times, sends him to a sick bed for a day or two; let that ailment depend upon well-known causes—causes that have been thoroughly considered by the patient himself, who has vainly tried to overcome them. Let it depend on hereditary defects, found in a special case, to be wholly incurable, and therefore to be simply endured, still, it matters not, the opinion is offered and insisted upon just the same. The friends or acquaintance who call upon him in his affliction withholding the same very rare genius, worthy of immortalization and embalment in your memory.

These meddlesome views of the laity upon matters of disease, in which they take upon themselves authority to advise a course of treatment, or to criticize the patient's habits of life, are often amusing, sometimes harmful, generally impertinent. It matters not to them whether the patient had sought and secured the best medical advice of both continents upon his case, and was carrying out the instructions thus obtained; or if the patient himself should chance to be a physician, more interested in his illness than any one else possibly could be, having studied his symptoms and read all the medical literature within his reach bearing upon his case, the advice comes just as promptly and just as impertinently.

Should the unfortunate invalid chance to attend a dinner party on the first day of the month, and have an attack of his malady on the thirty-first, the very wise dealer in advice and opinions consoles himself with the reflection, audibly expressed, that you are an ass for having consumed that dinner when you might have known it would have made you sick. Abstain from the dinner and remain quietly at home, then, you are accused of not eating enough and of remaining too steadfastly within doors. You are surprised to find that you ride out too much in the cold, and that you sit, by far, too continuously in your office. You read extravagantly, and yet play too much at cards, by the lattter amusement not sufficiently exercising your brain that it may the better perform its normal, healthy function. You drink too much wine, smoke too many cigars, partake too freely of strong coffee, yet permit your strength to wane for the lack of stimulants. You walk too much, or not enough. Have too much and too little company. But above all, you work far beyond your strength. Pshaw! Do you know that not one individual in a hundred—we meant to have said a thousand, but will keep it within bounds—who accuses himself of over-work, really ever does abuse himself in that manner?

The claim of "overwork" is a delicate compliment paid to the lazy sick man, but more frequently one usurped by himself. We always have our suspicions of a chap who compliments himself with the accusation of "overwork." Of course there *are* those who cripple their capability for physical endurance by engaging in brain and desk work too many hours continuously, without stopping long enough for meals and recreation. But the man who commences his office work at eight o'clock in the morning, takes an hour for luncheon at noon, and quits work at five in the afternoon, for dinner, returning no more to his office, does *not* work too hard. Of course it is understood that he must be in fairly good health to start with. Work is always a healthful, needful stimulant to all men. The man who does not use his brain and muscles in some useful employment, is a hindrance to himself and to his fellows. Business men will generally tell you that they never feel so well as when constantly engaged during every moment of their allotted working hours. It is a great mistake, therefore, to account for sickness, in the majority of instances, by setting up a plea of overwork.

But it is also a greater mistake to be striving continually to account for your neighbor's illness. It is not amusing, nor even triflingly entertaining to him to have you instruct him with regard to what your opinion of his case is, for he don't care a cent what you think, and does not want your confounded advice anyway. Let him alone. If he is fool enough to eat immoderately of late suppers, drink whiskey-cocktails and smoke strong cigars, knowing that they do him harm, and experiencing an attack of his trouble every time he so indulges and abuses himself, it is useless to talk to or advise such a man. Let him suffer the consequences of his foolishness—and he will be sure to, tenfold more than even your irritating lecture can inflict.

We are sorry for the man who, willing and anxious to work, is hindered with any sort of frequently occurring sickness, that interrupts his labors for a day or two. He has our unbounded sympathy and commiseration when his friends strive to point out the causes therefor.

---

DANGEROUS PORK.—At the village of Andover, in this state, occurred, recently, a case of trichina poisoning, in which a family of five persons were diseased through eating ham, of their own raising and curing. At this writing, all the members of the family are very seriously ill, and one of the children dead. We procured specimens of the ham, through the kindness of Dr. W. W. Crandall, the attending physician, and subjected it to microscopical examination. In one of the specimens, twelve mature worms were counted in a piece of the pork exactly one-eighth of an inch square and one-fiftieth of an inch thick. It will be seen that it requires a very small fragment of infected pork to produce sufficient numbers of the worms that cause the disease known as trichinosis. One mature female trichina will give birth to from one to six hundred young, when lodged in the stomach or intestines of the human being; therefore, when a full meal is made upon the infected ham, millions of the worms are set at liberty to invade the human system. When the pork containing the encysted trichina, is taken into the human stomach, the enveloping membrane, in which the worm is curled up, or "encysted," as it is termed, is dissolved, the worm set at liberty, and the young immediately developed, discharged alive, from the body of the parent, into the intestinal canal, and within three days commence their migrations through the intestinal walls, toward the muscular system. When they reach the muscles, in legs, arms, back and other portions of the body, they curl themselves up, an investing membrane is formed about them, and they become encysted, as when in the pork. If the patient is able to stand the pain and prostration caused during this migration period—lasting from six to ten days—he is apt to recover, with an enfeebled muscular system, resembling paralysis, due to the presence of the worms in the muscular fibre. Usually, however, the pain is so great and the nervous prostration so pronounced, that death ensues as a consequence.

The worms having become quiet, in their nests in the muscles, remain there, producing no further symptoms, and take on no evidences of life until they are set at liberty within the body or intestines of some host, when the same formula is gone through with as that which obtained in the case of their parents.

Recently, desiring a fragment of pork to compare with one of beef, to ascertain if any difference existed in the number of striæ to the inch—as seen under the microscope—we procured a specimen from one of our markets in this city, from a fine looking porker exposed for sale. The very first fragment we examined contained an encysted trichina!

The hog certainly is a very dangerous if not an uncleanly article of diet. If people *will* persist in eating "ham and eggs," be sure that the hog is well and thoroughly done!

The curing—salting and smoking of the hams—does not kill the encysted trichina. Their cell walls seem to be a complete protection to them, resisting the salt, and in many instances heat to the amount of 212°. Even thorough cooking is no certain peotection against them. The only infallible method of resisting their ravages that we have ever discovered, and can recommend as being absolutely safe, is found in these three words:—

Don't eat pork!

---

THE GERMAN METHOD OF WRITING.—A certain class of writers in this country affect the German method of writing. This don't refer to the peculiarly execrable caligraphy of our Teutonic brethren which, aided and assisted by their equally execrable printed letters, has made the Germans a nation of myopes, and promises, if not abandoned, to make them in the course of a generation or two, a nation of blind men, but to the scarcely less exasperating grammatical construction of sentences. Great processions of words unbroken by a period, and verbs divorced from their nouns and transported far, far away to the end of an interminable string of words; obscure, unnecessary and impertinent parenthetical clauses intruded at every possible opportunity under the pretense of making the meaning more clear, but with affect of hopelessly obscuring it; every device and expedient which can aid us in making the meaning obscure and the construction clumsy.

Why any one born to the noble inheritance of the English language should be willing to deface and defile it with idioms and modes of expression which are not only foreign to its genius, but in themselves inelegant and cumbrous, seems almost incomprehensible, unless, indeed, in the case of some crabbed dyspectic like the late Mr. Carlyle, who may select this method of venting his spleen upon his unoffending fellow countrymen, or some literary "dude" whose chief ambition is to make folks appreciate the momentous fact that he has been "abroad" and is now ready to receive the homages of an untraveled and untutored populace as the highest product of "foreign culture."

